# Therapeutic Potential of Sclareol in Experimental Models of Rheumatoid Arthritis

**DOI:** 10.3390/ijms19051351

**Published:** 2018-05-03

**Authors:** Sen-Wei Tsai, Ming-Chia Hsieh, Shiming Li, Shih-Chao Lin, Shun-Ping Wang, Caitlin W. Lehman, Christopher Z. Lien, Chi-Chien Lin

**Affiliations:** 1 Department of Physical Medicine and Rehabilitation, Taichung Tzu Chi Hospital, Buddhist Tzu Chi Medical Foundation, Taichung 427, Taiwan; tsaisenwei@gmail.com; 2 Department of Physical Medicine and Rehabilitation, School of Medicine, Tzu Chi University, Hualien 970, Taiwan; 3 Division of Endocrinology and Metabolism, Department of Internal Medicine, Changhua Christian Hospital, Changhua 500, Taiwan; mingchia570531@gmail.com; 4 Hubei Key Laboratory of Processing and Application of Catalytic Materials, College of Chemical Engineering, Huanggang Normal University, Huanggang 438000, China; shiming3702@gmail.com; 5 National Center for Biodefense and Infectious Diseases, School of Systems Biology, George Mason University, Manassas, VA 20110, USA; slin20@gmu.edu (S.-C.L.); cwoodso2@gmu.edu (C.W.L.); 6 Institute of Biomedical Science, National Chung-Hsing University, Taichung 40227, Taiwan; 7 Department of Orthopaedics, Taichung Veterans General Hospital, Taichung 40705, Taiwan; wsp0120@yahoo.com.tw; 8 Biodefense Program, Schar School of Policy and Government, George Mason University, Fairfax, VA 20110, USA; christopher.zane.lien@gmail.com; 9 Department of Medical Research, China Medical University Hospital, Taichung 40402, Taiwan

**Keywords:** sclareol, rheumatoid arthritis, synovial cell, collagen, mice, cytokines, Th17, MAPK

## Abstract

Previous studies have shown that the natural diterpene compound, sclareol, potentially inhibits inflammation, but it has not yet been determined whether sclareol can alleviate inflammation associated with rheumatoid arthritis (RA). Here, we utilized human synovial cell line, SW982, and an experimental murine model of rheumatoid arthritis, collagen-induced arthritis (CIA), to evaluate the therapeutic effects of sclareol in RA. Arthritic DBA/1J mice were dosed with 5 and 10 mg/kg sclareol intraperitoneally every other day over 21 days. Arthritic severity was evaluated by levels of anti-collagen II (anti-CII) antibody, inflammatory cytokines, and histopathologic examination of knee joint tissues. Our results reveal that the serum anti-CII antibody, cytokines interleukin (IL)-1β, IL-6, tumor necrosis factor (TNF)-α, and IL-17, as well as Th17 and Th1 cell population in inguinal lymph nodes, were significantly lower in sclareol-treated mice compared to the control group. Also, the sclareol treatment groups showed reduced swelling in the paws and lower histological arthritic scores, indicating that sclareol potentially mitigates collagen-induced arthritis. Furthermore, IL-1β-stimulated SW982 cells secreted less inflammatory cytokines (TNF-α and IL-6), which is associated with the downregulation of p38-mitogen-activated protein kinase (MAPK), extracellular signal-regulated kinase (ERK), and NF-κB pathways. Overall, we demonstrate that sclareol could relieve arthritic severities by modulating excessive inflammation and our study merits the pharmaceutical development of sclareol as a therapeutic treatment for inflammation associated with RA.

## 1. Introduction

Rheumatoid arthritis (RA) is an autoimmune disease characterized by synovial hyperplasia, chronic joint inflammation, and bone destruction, where fibroblast-like synoviocytes (FLS) appear to play a vital role in the in the pathogenesis of destructive arthritis [1]. The pathogenicity of RA exacerbated by FLS is attributed to the production of a wide range of cytokines and mediators, especially IL-6 and prostanoids, when activated by macrophage-like cells migrating from bone marrow to the synovium [2]. Specifically, RA-FLS can interact with other immune cells, including macrophages, dendritic cells, and lymphocytes, and disrupt immune homeostasis and create an inflammatory environment in the synovium, which contributes to cartilage and joint damage [3,4]. In addition, FLS can aggravate the progression of RA by secreting a number of pro-inflammatory cytokines, such as IL-6, IL-1β, TNF-α, and matrix metalloproteinases (MMPs, a matrix-degrading enzyme), and cause extracellular matrix (ECM) destruction [5,6].

Labdane diterpenes, also known as labdane-like bicyclic diterpenes, are a group of natural products sharing the same structural core and are prolific in various plants, such as Clary sage in our study. A wide variety of biological activities in labdane diterpenes have been identified, such as antimicrobial, antifungal, anti-inflammatory, and immunomodulatory functions [7,8,9,10]. Sclareol (labd-14-ene-8, 13-diol) is a member of bioactive labdane-type diterpenes, extracted from the leaves and flowers of Clary sage (*Salvia sclarea* L.) of the Lamiaceae family, one commonly cultivated for its essential oil that has been widely used as raw material for food, cosmetic products, and folk medicine. Several studies, both in vitro and in vivo, have shown that sclareol possesses immuno-modulation activities. For example, sclareol exhibits anti-inflammatory effects in lipopolysaccharide-stimulated RAW246.7 macrophages and in the λ-carrageenan-induced paw edema model via reducing expression of inducible nitric oxide synthase (iNOS) and cyclooxygenase-2 (COX-2) proteins [11]. More recently, sclareol was found to ameliorate lipopolysaccharide-induced pulmonary inflammation through the inhibition of NF-κB and MAPK and induction of heme oxygenase-1 (HO-1) signaling pathways [12]. Furthermore, sclareol exerts anti-osteoarthritic activities by regulating the balance between MMPs and TIMPs (tissue inhibitors of metalloproteinases) as well as inhibiting iNOS and COX-2 expression in interleukin-1β-induced rabbit chondrocytes and an experimental rabbit knee osteoarthritis model [13].

With its anti-inflammatory and immunomodulatory properties, sclareol is a promising candidate as an RA remedy agent. Therefore, the aims of this study were to determine and investigate the anti-arthritic activities of sclareol in a collagen-induced arthritis mouse model and SW982 human synovial cell line in order to evaluate the therapeutic potential of sclareol in treating RA.

## 2. Results

### 2.1. Amelioration of CIA by Sclareol Treatment

**Figure 1:**
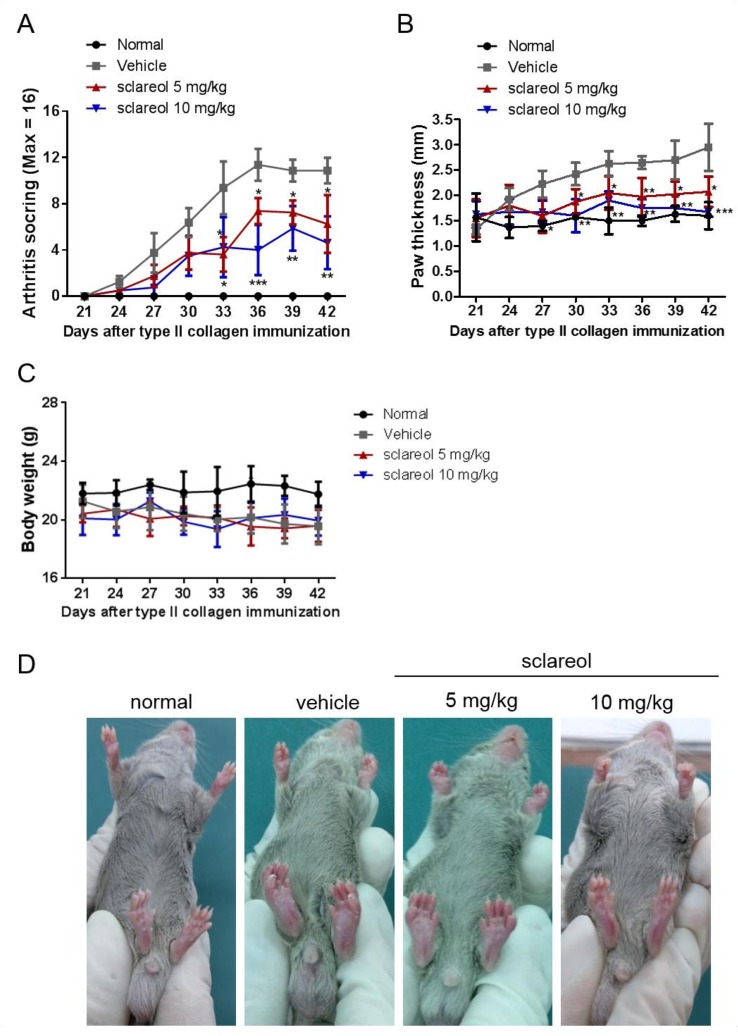
The effects of sclareol on the severity of collagen-induced arthritis (CIA). Mice with CIA were treated with 5 or 10 mg/kg of sclareol or vehicle every other day after arthritis onset on day 21. (**A**) The arthritis scores in each treatment group were monitored after booster immunization. (**B**) Paw swelling was measured by microcalipers, and the width of the hind paw for each mouse was averaged. Each point on the graph represents the mean ± standard deviation (SD) of six mice. (**C**) Body weight changes were monitored every 3 days after immunization with type II collagen (CII). (**D**) Photograph type (hind paw volume). The presented data are from a representative experiment that was repeated three times with similar results. * *p* < 0.05, ** *p* < 0.01, and *** *p* < 0.001 versus vehicle-treated CIA group.

**Figure 2:**
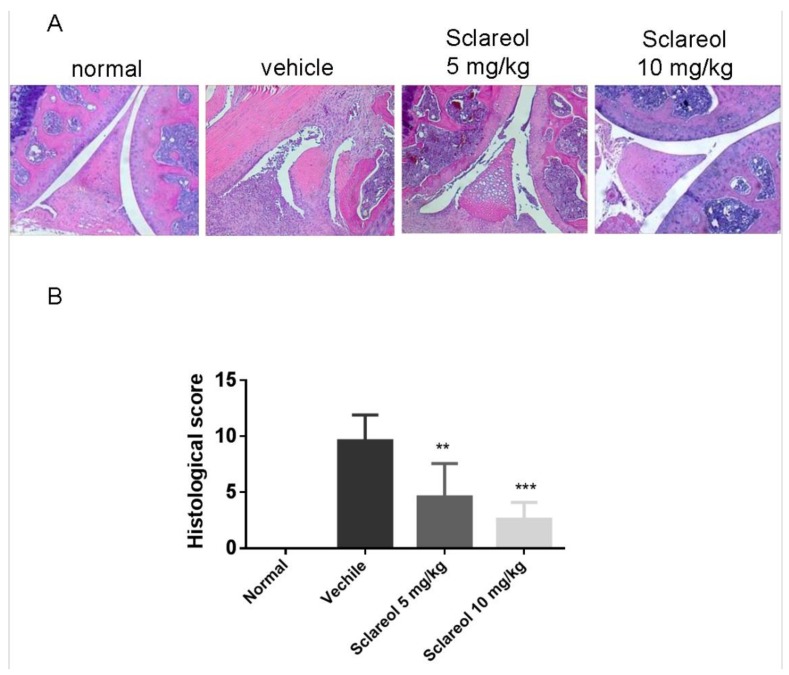
Histologic analysis of knee joints in mice on day 42. (**A**) Paraffin-embedded knee sections were stained with hematoxylin and eosin. Original magnification 100×. (**B**) The degrees of joint damage were scored with or without sclareol treatments. Data expressed as means ± SD of six mice in each group. The presented data are from a representative experiment that was repeated three times with similar results. ** *p* < 0.01 and *** *p* < 0.001 versus vehicle-treated CIA group.

To determine the anti-arthritic effects of sclareol, we examined collagen-induced arthritis (CIA) progression in DBA/1J mice. On day 21 after primary immunization with CIA, when the clinical signs of arthritis first appeared, mice were intraperitoneally treated with either a daily administration of sclareol (5 and 10 mg/kg) or with a vehicle control for another 21 days. We first confirmed that the arthritic scores in CIA mice were significantly increased compared to that of the non-immunized mice throughout the experiment (Figure 1A). Mice receiving 5 and 10 mg/kg sclareol intraperitoneally displayed profound reductions in clinical scores compared to vehicle control mice. Similarly, 5 and 10 mg/kg sclareol-treated mice had reduced paw swelling compared to controls (Figure 1B,D). Histologically, the knee joints of vehicle-treated mice displayed notable synovial hyperplasia, high numbers of inflammatory cytokines, and severe cartilage damage and bone erosions. Conversely, sclareol-treated groups exhibited substantially alleviated clinical symptoms (Figure 2 and Supplementary Figure S1), suggesting that sclareol mitigates arthritic progression in our CIA mouse model, enhancing alleviation of inflammatory arthritis. Of note, sclareol treatments did not cause behavioral abnormalities or significant body changes in CIA mice (Figure 1C), whose average body weights are slightly lower than normal mice, indicating that administration of sclareol at 5 and 10 mg/kg does not induce toxicity.

### 2.2. Decreased Levels of Circulating Anti-CII Abs and Cytokines in Sclareol-Treated CIA Mice

**Figure 3:**
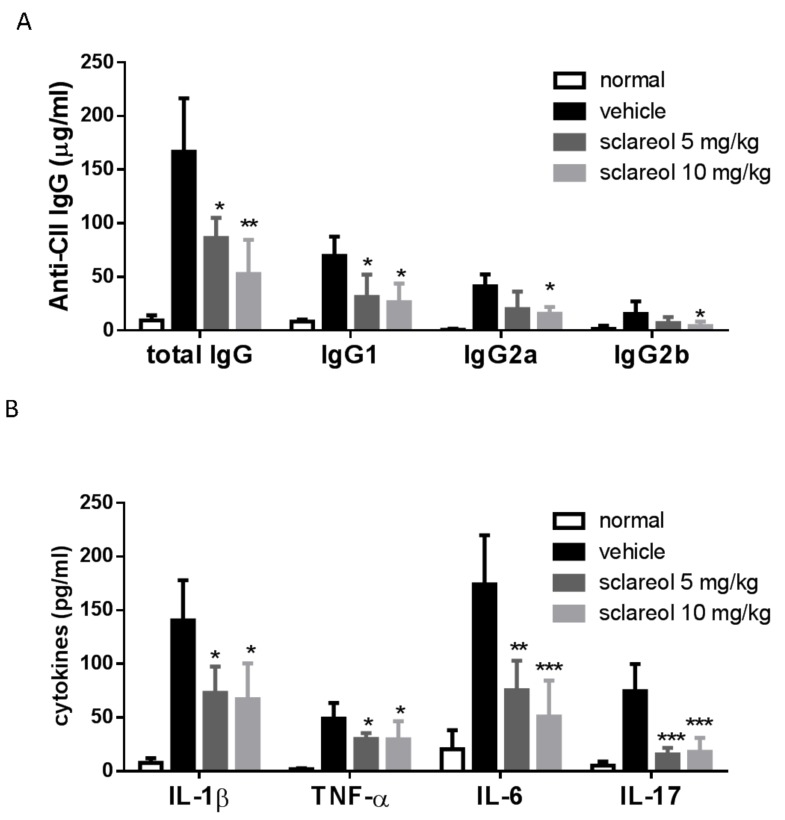
Effects of sclareol on anti-collagen II (anti-CII) IgG specific autoantibodies and serum cytokine levels. Serum obtained from each group on day 42 was measured for (**A**) anti-mouse collagen II IgGs and (**B**) pro-inflammatory cytokines by ELISA. Values on the graph represent the means ± SD from six mice/group. * *p* < 0.05, ** *p* < 0.01, and *** *p* < 0.001 versus vehicle-treated CIA group.

We next explored the effect of sclareol on serum levels of anti-collagen II (anti-CII) Abs, which play an important role in the pathogenesis of CIA [14]. As shown in Figure 3A, levels of total IgG, IgG1, IgG2a, IgG2b antibodies in the serum of sclareol-treated mice were markedly reduced compared to vehicle-treated mice.

Multiple proinflammatory cytokines, such as IL-1β, IL-6, TNF-α, and IL-17, cause cartilage damage and bone destruction in aggravation of rheumatoid arthritis [15]. Thus, the regulation of these cytokines may be an appropriate approach to manage the development and progression of RA. Based on this concept, we detected the concentrations of the inflammatory cytokines in serum on day 42 by enzyme-linked immunosorbent assay (ELISA). Results (Figure 3B) reveal that compared to vehicle-treated control mice, IL-1β, IL-6, TNF-α, and IL-17 in the serum from sclareol-treated groups were markedly decreased.

Our data not only confirm that the concentrations of anti-CII antibodies and pro-inflammatory cytokines are elevated in CIA mice, which is consistent with previous works [14,15], but also imply that the humoral immune response and these pro-inflammatory cytokines could be involved in sclareol-mediated modulation of inflammation in the CIA model of inflammatory arthritis.

### 2.3. Altered Frequency of Th17 and Th1 Cells in Lymph Nodes by Sclareol Treatment

**Figure 4:**
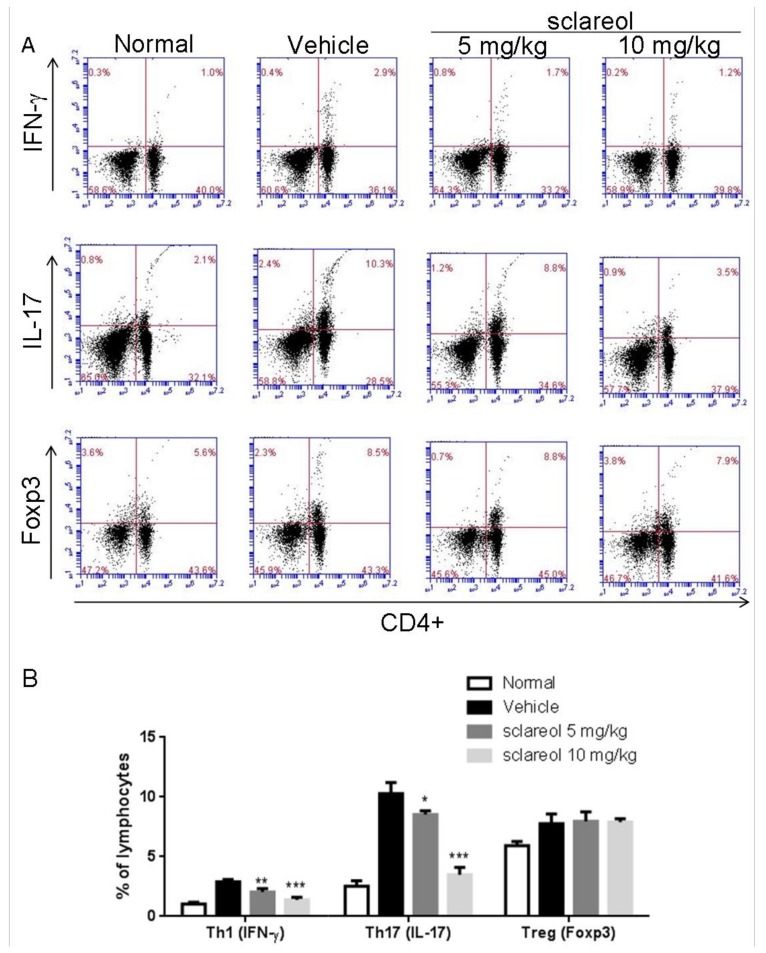
Sclareol reduced Th1 cells (CD4^+^ IFN-γ^+^ T cells) and Th17 cells (CD4^+^ IL-17^+^ T cells) in the inguinal lymph nodes (ILNs). (**A**) Single cell suspensions were collected from ILNs, followed by stimulation with 20 ng/mL phorbol myristate acetate (PMA) and 1 μg/mL ionomycin in the presence of 10 μg/mL brefeldin A for 4 h and then stained with anti-CD4, anti-IFN-γ, anti-IL-17A, or anti-Foxp3 Abs and analyzed by flow cytometry. Representative results in each group are shown. (**B**) Cell population results were quantified and represent as the mean ± SD with six mice per group. * *p* < 0.5, ** *p* < 0.01 and *** *p* < 0.001 versus vehicle-treated CIA group.

The dynamic of Th17 and regulatory (Treg) populations in peripheral blood is implicated in the pathogenesis of RA [16], and Th1 responses have also been shown to be predominant in CIA mice [17]. To elucidate whether Th17/Treg and Th1 are associated with the sclareol-mediated anti-arthritic activity, we examined the frequency of Th17, Th1, and Treg cells in draining inguinal lymph nodes (ILNs) using flow cytometry. As shown in Figure 4, the levels of CD4^+^ IL-17^+^ Th17 and CD4^+^IFN-γ^+^ Th1 cells, but not FOXP3^+^ CD4^+^ regulatory T cells, isolated from sclareol-treated CIA mice were lower than those in vehicle-treated CIA mice. This indicates that the sclareol treatment could exert its anti-arthritic effects via decreasing the Th17 and Th1 cell populations, but not increasing Treg cells, suggesting the change in Th17/Treg and the decrease of Th1 could be involved in mitigating the collagen-induced arthritis in sclareol-treated mice.

### 2.4. Cytotoxicity of Sclareol on Synovial Cells In Vitro

**Figure 5:**
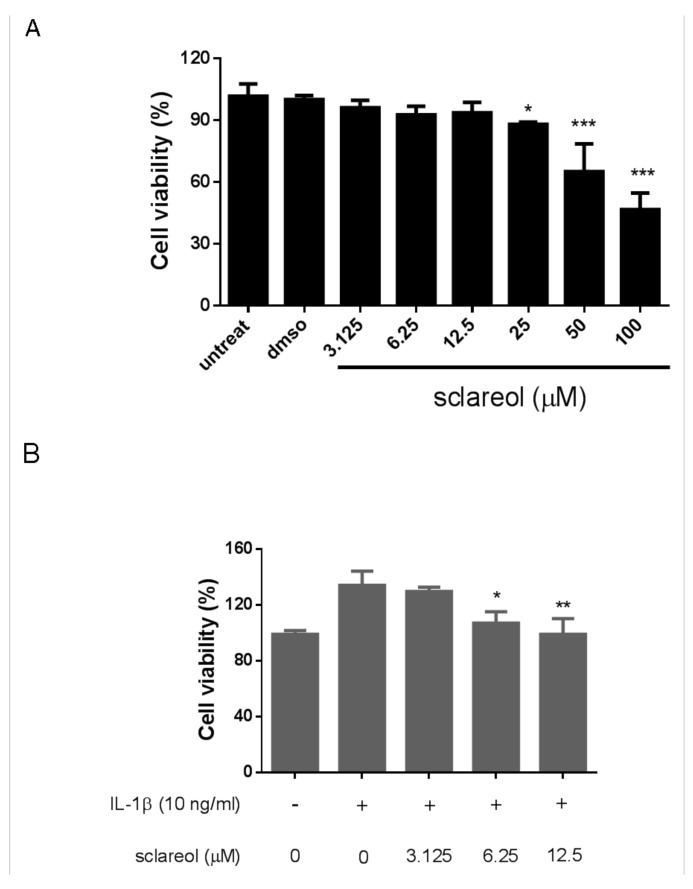
Effects of sclareol on SW982 cell viability. (**A**) The cytotoxicity of sclareol to SW982 cells was evaluated with the CCK-8 assay in the absence of IL-1β. (**B**) The cell viability of SW982 cells treated with IL-1β for 72 h was monitored. Each bar on the bar graph represents the mean ± SD of triplicate tests. The data are representative of three independent experiments with similar results. * *p* < 0.05, ** *p* < 0.01, and *** *p* < 0.001 versus vehicle treated dimethyl sulfoxide (DMSO) group.

In order to have a better understanding of the effects of sclareol on RA, we next utilized a synovial cell line, SW982, as a model to investigate the interaction between FLS and sclareol. We added various doses of sclareol (from 3.125 to 100 μM) to SW982 cells for 72 h and measured cell viability by CCK-8 assay. As shown in Figure 5A, sclareol had very limited cytotoxic effects on SW982 cells in concentrations between 3.125 to 12.5 μM but not concentrations ≥25 μM. As a result, we only selected 6.25 and 12.5 μM for the following experiments. Consistent with the results from a previous study [18], treatment with IL-1β (10 ng/mL) to SW982 cells for 72 h markedly increased cell viability and proliferation, thus confirming that synovial fibroblasts, like SW982 cells, could indeed proliferate after IL-1β exposure and might play a role in the pathogenesis of RA. However, the proliferation of SW982 cells was reversed with treatment of sclareol in a dose-dependent manner (Figure 5B), indicating that sclareol significantly reduced IL-1β-induced SW982 cell viability. Notably, treatment with sclareol at the same concentrations in the absence of IL-1β did not affect cell proliferation (Figure 5A). Similarly, we also observed reversed proliferation with sclareol treatment in IL-1β-treated human primary synoviocytes (Supplementary Figure S2A).

### 2.5. Sclareol Downregulated Interleukin-1β-Induced Expression of Matrix Metalloproteinases and Proinflammatory Cytokines in Synovial Cells

**Figure 6:**
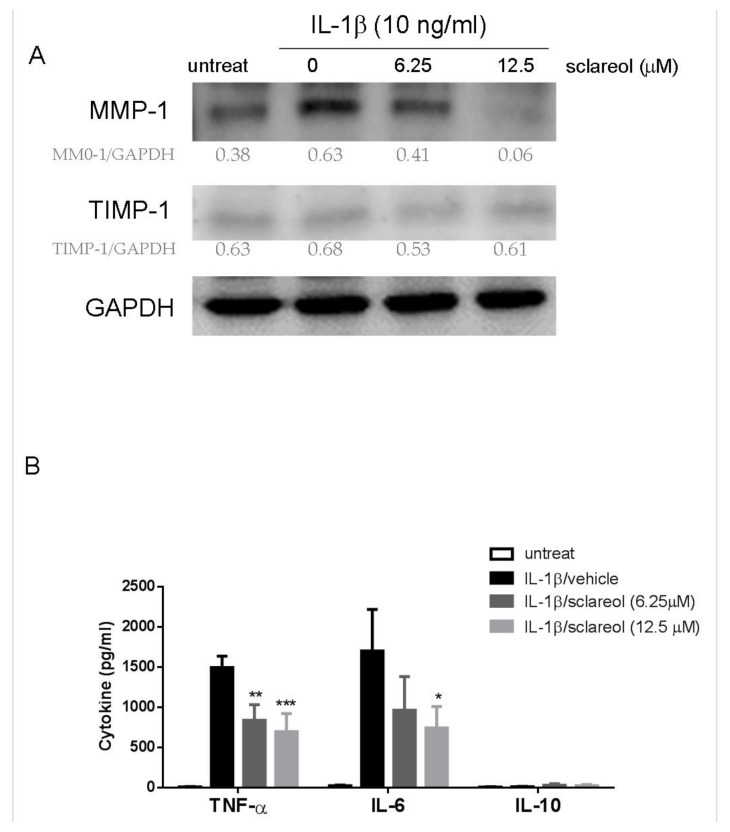
Effect of sclareol on IL-1β-induced production of matrix metalloproteinases (MMPs) and cytokines in SW982 cells. SW982 cells were stimulated with 10 ng/mL IL-1β with or without for sclareol for 72 h. (**A**) Expressions of MMP-1 and tissue inhibitors of metalloproteinase (TIMP)-1 in whole cell lysates were determined by Western blot with indicated antibodies. Glyceraldehyde 3-phosphate dehydrogenase (GAPDH) was used as a loading control, the normalized values (mean ± SD) compared to GAPDH were estimated by Image J software, and the normalized values were attached to each photographic band in the images. (**B**) The cytokine levels in culture supernatant were examined by ELISA. Bar graphs represent the mean ± SD of triplicate tests. * *p* < 0.05, ** *p* < 0.01, and *** *p* < 0.001 versus vehicle treated DMSO group. The data are representative of three independent experiments with similar results.

IL-1β is considered one of the key cytokines involved in the pathogenesis of RA, and SW982 cells reportedly produce matrix metalloproteinase (MMP) and inflammatory cytokines in response to IL-1β, which markedly resemble the inflamed synovial tissue associated with RA [19]. Here, we stimulated SW982 cells with IL-1β (10 ng/mL) in the presence or absence of sclareol for 72 h. Western blotting assays were used to detect the expressions of MMP-1 and tissue inhibitor of metallopeptidase 1 (TIMP-1) proteins in SW982 cells. Data in Figure 6A shows that 12.5 μM of sclareol decreased the expression of IL-1β-induced MMP1, but not TIMP-1, which suggests that sclareol suppresses the degradation of extracellular matrix (ECM) caused by RA. We also investigated the effect of sclareol on the proinflammatory cytokines IL-6 and TNF-α produced by IL-1β-stimulated SW982 cells and human primary synoviocytes. The ELISA results shown in Figure 6B and Figure S2B reveal that sclareol significantly decreased IL-1β-induced TNF-α and IL-6 expression in both cells but not the anti-inflammatory cytokine IL-10 expression. Thus, our results demonstrate that sclareol can suppress inflammatory effects on IL-1β-treated synovial cells.

### 2.6. Sclareol Modulates IL-1β-Induced MAPK and NF-κB Pathways in SW982 Cells

**Figure 7:**
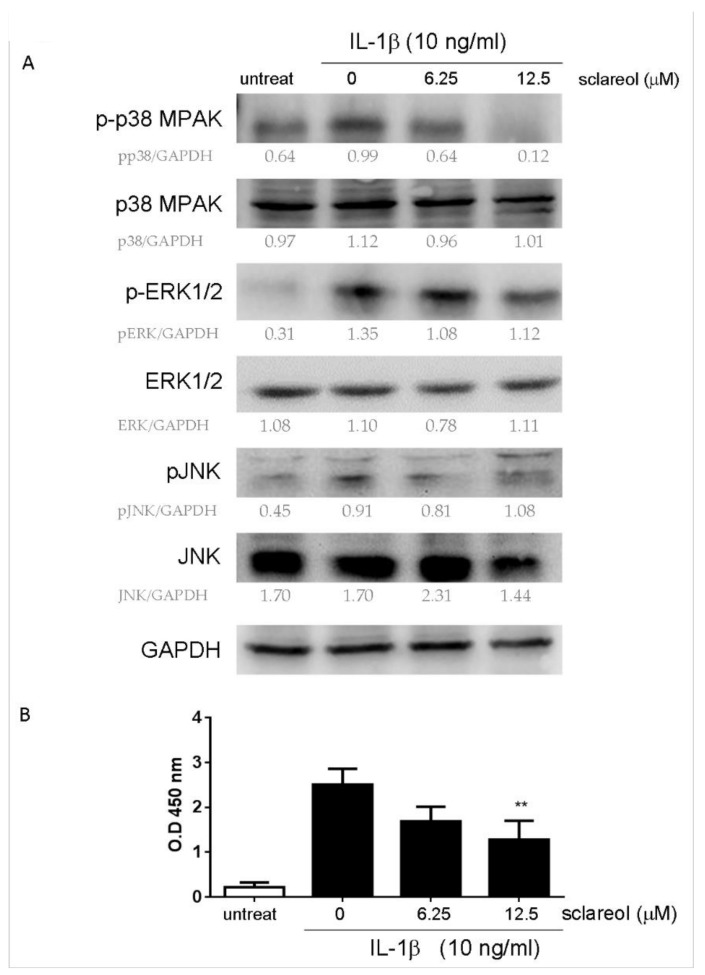
Effect of sclareol on MAPK and NF-κB pathways in IL-1β-stimulated SW982 cells. SW982 cells were stimulated with 10 ng/mL of IL-1β with or without sclareol and lysed after 24 h. (**A**) Expressions of c-Jun N-terminal kinase (JNK), ERK, and p38-MAPK (native and phosphorylated) in whole cell lysates were determined by Western blot with the indicated antibodies. GAPDH was used as a loading control. (**B**) The translocational activities of NF-κB with or without sclareol were determined by NF-κB activation and presented as optical density (OD) values at a wavelength of 450 nm. Bar graphs represent the mean ± SD of triplicate tests. ** *p* < 0.01 versus vehicle-treated DMSO group. The data are representative of three independent experiments with similar results.

As shown previously, the activation of MAPKs and NF-κB plays a pivotal role in the production of cytokine and MMPs by synovial fibroblasts in response to inflammatory stimuli, like IL-1β and TNF-α. To gain insight into the mechanism of inhibitory action of sclareol, we examined the protein levels of MAPKs (p38, extracellular signal-regulated kinase (ERK) -1/2, c-Jun N-terminal kinase (JNK)) and NF-κB activation in IL-1β-stimulated SW982 cells after sclareol treatments. As shown in Figure 7A, the phosphorylations of ERK and p38 induced by IL-1β were attenuated in the presence of sclareol. Furthermore, we measured the nuclear translocation of NF-κB by using the TransAM NF-κB transcription factor assay. The NF-κB binding activity was upregulated following 24 h of IL-1β stimulation, however, upon treatment with sclareol (12.5 μM), IL-1β-induced NF-κB binding activity was significantly prevented (Figure 7B). Together, these results indicate that sclareol might achieve its anti-inflammatory effects via suppressing the MAPK and NF-κB pathways in IL-1β-induced SW982 cells.

## 3. Discussion

The data we show in this study have provided evidence to demonstrate that the natural diterpene, sclareol, could significantly diminish over-reactive systemic inflammation and humoral immunity in the CIA mouse model, which is characterized by lower pro-inflammatory cytokines such as IL-1β, IL-6, TNF-α, and IL-17 as well as reduced serum anti-CII antibodies. Also, sclareol treatment reduced Th17 cells from inguinal lymph nodes (ILNs) of CIA mice, contributing to the alleviation of arthritic symptoms such as reduced swelling in paws and less synovial hyperplasia in joints. We further demonstrated that sclareol treatment inhibits pro-inflammatory cytokine production from IL-1β-stimulated SW982 human synovial cells, possibly through the downregulation of MMP-1, the suppression of p38MAPK and ERK1/2 signaling pathways, and NF-κB translocation.

Sclareol is one of the main components present in the essential oil extracted from Clary sage (*Salvia sclarea* L.). Traditionally, *S. sclarea* oil has been used in herbal medicine for pain-relieving and anti-spasmodic activities [20]. More recently, sclareol was identified as an active constituent from the calyces of *S. sclarea*, contributing to the various bioactivities in *S. sclarea* [21], in particular, anti-inflammation. Sclareol has been shown to attenuate the lipopolysaccharides (LPS)-induced paw edema and pulmonary injury in mice, which justified the rationale of this study to test sclareol in arthritis [11,12]. Of note, our study is not the first report to identify the anti-arthritic function of sclareol. Zhong, Y. et al. reported that sclareol mitigated osteoarthritis in an IL-1β-stimulated rabbit model [13], but the cytokine, humoral, and cell-mediated immune responses after treatment were not investigated, whereas our study addressed this issue.

The roles of cytokines and T lymphocyte subpopulations in the pathogenesis of RA have been gradually clarified. Recent reports have implied that pathogenic Th17 cells and IL-17 mediate pannus growth [22], osteoclastogenesis [23], and synovial neoangiogenesis, explaining the severity of symptoms in RA patients [24]. Moreover, the imbalance of Th17/Treg appears to exacerbate the symptoms of RA [16]. We observed an elevated serum level of IL-17 and increased frequencies of IL-17-producing T helper (Th17) and Treg cells in the inguinal lymph nodes of CIA mice. The frequency of IL-17 and Th17 cells were decreased with the administration of sclareol, but there were no significant changes in Treg cell populations among CIA mouse groups. We reasoned that the anti-arthritic effects of sclareol could be in favor of Th17-related pathways but not Treg. However, on the contrary, an observation from a cancer study indicated that sclareol reduced the number of splenic CD4^+^, CD25^+^, FoxP3^+^, and Treg cells in breast cancer mice [25]. Regardless of the differences between the two distinct disease models, it is obvious that the impact of sclareol on Treg cells needs to be further elucidated. Nevertheless, with more understanding of sclareol-inhibited Th17 and IL-17, sclareol could also be used in other immune-mediated illnesses such as systemic sclerosis or glomerular disease.

Increasing evidence shows that the activation of multiple stress signaling pathways induced by IL-1β or TNF-α (e.g., MAPKs and NF-κB) could potentially be associated with the pathogenic mechanisms of joint destruction and inflammation in RA [26,27]. Inhibitors of MAPK or NF-κB, such as bortezomib and cobimetinib, have also been shown to alleviate the synovial inflammation, bone destruction, and cartilage damage in CIA and adjuvant arthritis animal models [28,29]. Additionally, pro-inflammatory cytokines and MMP-1, which have both been verified as pathogenic factors in arthritic patients, were elevated in joint tissues in response to stimulation with transcription factor NF-κB and MAPK [30]. Therefore, the signaling cascades involving NF-κB or MAPKs are considered to be promising therapeutic targets for arthritis intervention. In the present study, sclareol appears to possess multiple facets of inhibitory function against arthritic inflammation. Sclareol not only ameliorated the histological destruction in synovial tissues but also suppressed the activation of the MAPKs and NF-κB translocation induced either by collagen or IL-1β, endowing sclareol with a new pivotal role in the combat of rheumatoid arthritis. Our results reveal that sclareol inhibits the IL-1β-induced phosphorylation and nuclear translocation of NF-κB p65 subunit, indicating the inhibiting potential of sclareol on inflammation-induced NF-κB phosphorylation in cultured FLS-like SW982 cells. Meanwhile, sclareol also significantly diminished IL-1β-triggered phosphorylation of ERK and p38, demonstrating the inhibitory effects of sclareol on MAPK activation in synoviocytes.

In this study, we present the results of the interactions between sclareol and both human FLS cell types, SW982 and primary human synoviocytes, both of which exhibit similar patterns in terms of the anti-arthritic activity when treated with sclareol (Figure 5, Figure 6, and Figure S2). When selecting cellular material for study, there are always advantages and disadvantages that must be evaluated prior to finalizing study design. For example, the stable cellular status in transformed cells is the major advantage for assessing the anti-inflammatory activities of sclareol in vitro. Contrarily, the quality of primary cells is labile due to the variable and highly dependent nature of primary cells on physiological conditions of the patient. Additionally, the sensitivity of primary cells to cultural environment conditions, such as the growth factors and the following experimental procedures, must also be considered. These variables could lead to inconsistent results, possibly jeopardizing the conclusion of a study. However, to avoid the potential abnormal growth conditions of a sarcoma cell line, it would be rational and desirable to utilize primary synovial cells for investigation of the anti-inflammatory efficacy induced by treatment with sclareol.

In summary, our study demonstrates that sclareol can decelerate the IL-1β-induced expression of MMP-1, TNF-α, and IL-6 in SW982 cells via attenuating translocation of NF-κB and phosphorylation of MAPK pathways, including p-38 and ERK. Moreover, sclareol can remarkably improve clinical symptoms, such as paw swelling and bone erosions, and reduce the number of Th17 cells in CIA mice. These findings indicate the pharmacological potential of sclareol and provide a therapeutic direction for applying sclareol towards the clinical treatments for rheumatoid arthritis and other inflammatory diseases.

## 4. Material and Methods

### 4.1. Animal Experiments

Eight-week-old male DBA/1J mice (20–22 g in weight) were purchased from Jackson Laboratory (Bar Harbor, MA, USA) and kept under specific-pathogen-free (SPF) conditions with food and water ad libitum. All animals were treated in accordance with the Institutional Animal Care and Use Committee (IACUC) of National Chung Hsing University (NCHU), and the study protocols were approved by the Committee on Animal Research and Care in NCHU (NO. 104070). The animal model of collagen type II-induced arthritis (CIA) was used as described previously [31]. Sclareol was purchased directly from Sigma Aldrich Co. (St. Louis, MO, USA) and dissolved in the corn oil/DMSO vehicles (*v*/*v*, 95/5). Male DBA/1J mice were randomly divided into four groups of equal number (*n* = 6): (1) normal/control group; (2) the CIA + vehicle group; (3) the CIA + 5 mg/kg sclareol group; and (4) the CIA + 10 mg/kg sclareol group. The mice in groups 2, 3, and 4 received either vehicle or sclareol via intraperitoneal injection every other day from day 21 to day 42, whereas group 1 was given 100 μL corn oil/DMSO as a vehicle control. Disease severities of CIA mice were scored by clinical symptoms of limbs by two investigators in a blinded manner from day 21 post-immunization. Clinical arthritis scores from 0 to 4 were recorded based on swelling levels of paws measured with microcalipers, erythema, edema, and joint rigidity. The maximal arthritis score per paw is 4, where 0 means no swelling; each limb was graded, and therefore, a maximal score was 16 for each animal. Mice were sacrificed on day 42 post-immunization for further histological examination.

### 4.2. Histological Analysis

The knee joints were removed at the end of the experiments. Samples were fixed in 10% formalin decalcified with 15% ethylenediaminetetraacetic acid (EDTA) and embedded in paraffin for tissue sections (5 μM thick), which in turn were stained with hematoxylin and eosin (H & E) according to standard methods. Histopathological damage was blindly scored according to previously defined parameters [32]. In brief, cell infiltration, synovial hyperplasia, and cartilage destruction were assigned scores of 0–4 by a pathologist based on the following criteria: 0, no changes; 1, mild changes; 2, moderate changes; 3, severe changes; 4, total destruction of joint architecture. A value for each knee joint was obtained and yielded the maximum possible score of 8.

### 4.3. ELISA for Serum Anti-Mouse Collagen II Antibodies (Anti-CII Abs)

Anti-CII Ab ELISA kits were purchased from Chondrex (Redmond, WA, USA). Sera samples were collected from each mouse at the end of the experiment and the titers of anti-CII Abs (total IgG, IgG1, IgG2a, and IgG2b) were assessed following the manufacturer’s instructions. Tested sera were diluted 2500-fold and added to mouse CII-coated 96-well plates overnight at 4 °C. Bound IgG was detected by incubation with horseradish peroxidase (HRP)-conjugated anti-mouse IgG, followed by o-Phenylenediamine (OPD) substrate. Serum cytokines were measured by standard sandwich ELISA according to the manufacturer’s protocol, eBioscience Co., Ltd. (San Diego, CA, USA).

### 4.4. Flow Cytometry

Intracellular cytokine staining and flow cytometry were modified from our previous report [30]. Briefly, on day 42, single cell suspensions from inguinal lymph nodes (ILNs) were pulsed with 20 ng/mL PMA (Sigma-Aldrich, St. Louis, MO, USA) and 1 μg/mL ionomycin (Sigma-Aldrich, St. Louis, MO, USA) for 18 h, with 10 μg/mL brefeldin A was added during the last 4 h of culture. After stimulation, the cells were surface stained with phycoerythrin- (PE-) anti-CD4 antibody (BD Biosciences, San Diego, CA, USA), permeabilized/fixed with cytofix/Cytoperm Plus (BD Biosciences), and stained with FITC-anti-IL-17A antibody and FITC-anti-IFN-γ antibody (Biolegend). To analyze regulatory T cells (Tregs), single cell suspensions from ILNs were stained with PE-anti-CD4 antibody (BD Biosciences), fixed, permeabilized, and stained with anti-Foxp3 antibody (BD Biosciences) according to the manufacturer’s instructions. Flow cytometer analysis was performed in an AccuriTM C5 cytometer.

### 4.5. Cell Culture

The SW982 human synovial cell line was purchased from the American Type Culture Collection (ATCC; Manassas, VA, USA) and was maintained in Leibovitz-15 medium with 10% fetal bovine serum (FBS), 100 U/mL penicillin, and 100 μg/mL streptomycin. The human synovial primary cell was purchased from Celprogen (36069-02, San Pedro, CA, USA) and was cultured in human synovial fluid membrane fibroblast primary cell culture complete media with serum (M36069-03S, Celprogen). Both cell lines were maintained at 37 °C under a humidified atmosphere containing 5% CO_2_ with medium changed every 3 days.

### 4.6. Cell Counting Kit-8 (CCK8) Assay

Cell viability was determined using the CCK8 assay (Dojindo, Kumamoto, Japan). In brief, the SW982 cells or human synovial primary cells were seeded in 96-well plates (Corning Costar, Corning, NY, USA) or extra-cellular matrix pre-coated 96 well plates (Celprogen, Torrance, CA, USA) at a density of 10^5^ cells per well in 100 μL medium, incubated overnight, and then treated with sclareol (3.125–100 μM) with or without IL-1β (10 ng/mL) for 72 h. The control wells contained an equivalent amount of medium containing 0.1% DMSO. CCK8 regent was added to each well, incubated for 1 h, and absorbance (optical density, OD) was determined using a microplate reader (Tecan Sunrise, Männedorf, Switzerland) at 450 nm.

### 4.7. Western Blot Analysis

SW982 cells (2 × 10^5^/well) were seeded into 6-well plates and treated with sclareol (6.25 or 12.5 μM) with or without IL-1β (10 ng/mL) for 24 h. The cells were then lysed in RIPA buffer (Sigma-Aldrich, St. Louis, MO, USA) containing 1% protease inhibitor cocktail (Sigma-Aldrich). Equal loading protein concentration was measured by using the BCA Protein Assay Kit (Thermo Fisher Scientific, Waltham, MA, USA). Proteins were separated by 10% sodium dodecyl sulfate polyacrylamide gel electrophoresis (SDS-PAGE), and then transferred to a polyvinylidene difluoride (PVDF) membrane. After blocking with 5% skim milk for 2 h at room temperature, the membranes were incubated with anti-MMP-1 (R & D systems, Minneapolis, MN, USA), anti-tissue inhibitor of metalloproteinase 1 (TIMP-1) (Sigma, St. Louis, MO, USA), anti-phospho-p38 (Thr180/Tyr182), anti-p38, anti-phospho-p42/44 (Thr202/Tyr204, 20G11), anti-total p42/44 (137F5), anti-phosphor-c-Jun N-terminal kinase (anti-phospho-JNK) (81E11), anti-JNK (all purchased from Cell Signaling, Danvers, MA, USA) and glyceraldehyde 3-phosphate dehydrogenase (GAPDH) (Cat# ab8245, Abcam, Cambridge, MA, USA) antibodies at 4 °C overnight. The membranes were then incubated with appropriate HRP-conjugated secondary antibodies (Jackson ImmunoResearch Laboratories, West Grove, PA, USA) overnight at 4 °C. The immunoactive bands were detected with an enhanced chemiluminescence (ECL) system and developed using the Hansor Luminescence Image System (Taichung, Taiwan). All bands in the blots were normalized to the level of GAPDH for each lane. The band density was measured with the ImageJ v1.47 program for Windows from the National Institute of Health (NIH) (Bethesda, Rockville, MD, USA).

### 4.8. NF-κB Activity Assay

The SW982 cells were harvested after 24 h treatment with sclareol, and nuclear extracts were prepared using the NE-PER Nuclear and Cytoplasmic Extraction system (Thermo Fisher Scientific, Waltham, MA, USA). For each assay, a total of 10 μg nuclear extract was used in a TransAM NF-κB p65 ELISA kit (Active Motif, Carlsbad, CA, USA) according to the manufacturer’s instructions.

### 4.9. Measurement of the Cytokine Concentrations in Cell Culture

After washing with PBS (pH 7.4), the SW982 or human synovial fluid normal membrane fibroblast primary cells (2 × 10^5^/well) were incubated at 37 °C with or without sclareol and IL-1β (10 ng/mL) in Leibovitz-15 medium containing 10% (*v*/*v*) FBS for 24 h. Culture supernatants were collected and stored at −80 °C. The cytokine (TNF-α and IL-6) concentrations in the medium were measured by standard sandwich ELISA according to the manufacturer’s protocol (eBioscience).

### 4.10. Statistical Analysis

Data were expressed as the mean ± SD. All statistical analyses were performed using either one-way ANOVA or two-way ANOVA with subsequent Tukey’s HSD (honest significant difference) test to compare multiple treatments using GraphPad Prism (version 5 for Windows; GraphPad Software, La Jolla, CA, USA). The significance of difference was defined as *p* values < 0.05.

## Supplementary Materials

Supplementary materials can be found at http://www.mdpi.com/1422-0067/19/5/1351/s1.
