# Supplementary material for: Therapeutic Potential of Sclareol in Experimental Models of Rheumatoid Arthritis
Source: Int J Mol Sci. 2018 May 3;19(5):1351. doi: 10.3390/ijms19051351 (PMC5983692; doi:10.3390/ijms19051351)
Supplement: Supplementary file 1 [file ijms-19-01351-s001.pdf]

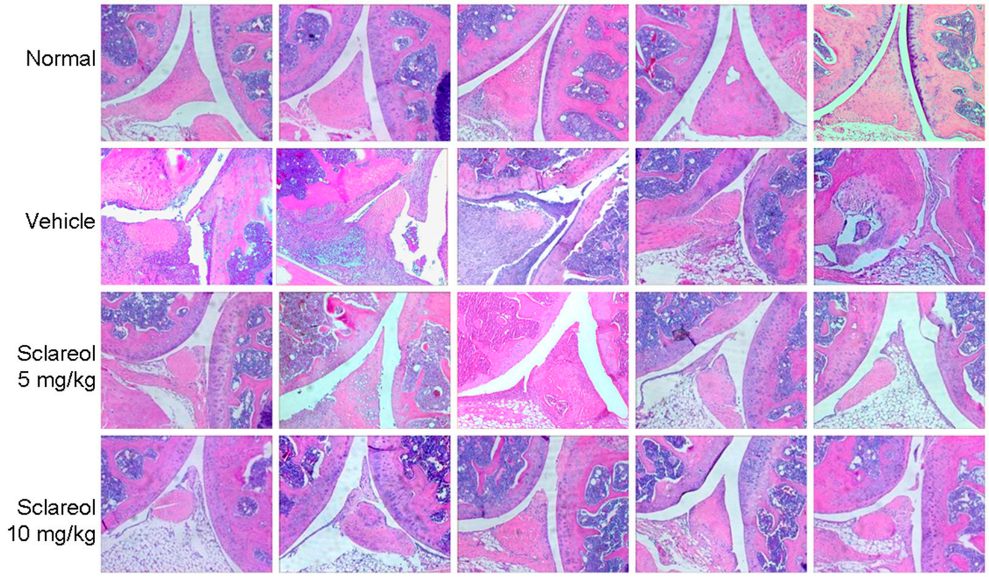

Supplementary Figure 1. Histologic analysis of knee joints in mice on day 42. (A) Paraffin-embedded knee sections were stained with hematoxylin and eosin. Original magnification ×100.

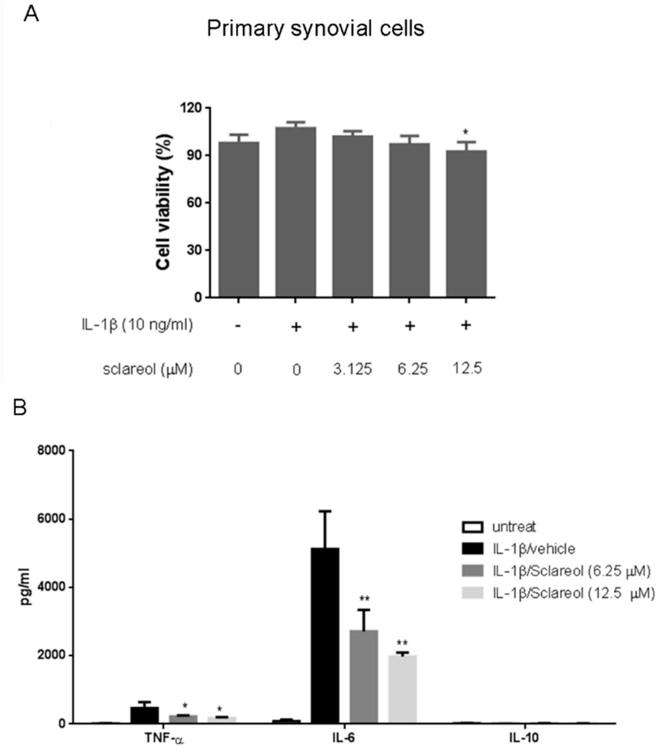

Supplemental Figure 2. Effect of sclareol on cell viability and cytokines production in human synovial fibroblast primary cells. Cells were stimulated with 10 ng/mL IL-1β with or without for sclareol for 72 h. (**A**) The cell proliferation of human synovial fibroblast primary cells was evaluated with the CCK-8 assay. (**B**) The cytokine levels in culture supernatant were examined by ELISA. Bar graphs represent the mean ± SD of triplicate tests. *$p < 0.05$ and **$p < 0.01$ versus DMSO-treated vehicle group. The data are representative of three independent experiments with similar results.
